# Disrupted Topology of Frontostriatal Circuits Is Linked to the Severity of Insomnia

**DOI:** 10.3389/fnins.2017.00214

**Published:** 2017-04-19

**Authors:** Feng-Mei Lu, Chun-Hong Liu, Shun-Li Lu, Li-Rong Tang, Chang-Le Tie, Juan Zhang, Zhen Yuan

**Affiliations:** ^1^Bioimaging Core, Faculty of Health Sciences, University of MacauMacau, China; ^2^Beijing Hospital of Traditional Chinese Medicine, Capital Medical University, Beijing Institute of Traditional Chinese MedicineBeijing, China; ^3^Department of Radiology, Beijing Anding Hospital, Capital Medical UniversityBeijing, China; ^4^Faculty of Education, University of MacauMacau, China

**Keywords:** insomnia, resting-state fMRI, graph theory, salience network, frontostriatal connectivity

## Abstract

Insomnia is one of the most common health complaints, with a high prevalence of 30~50% in the general population. In particular, neuroimaging research has revealed that widespread dysfunctions in brain regions involved in hyperarousal are strongly correlated with insomnia. However, whether the topology of the intrinsic connectivity is aberrant in insomnia remains largely unknown. In this study, resting-state functional magnetic resonance imaging (rsfMRI) in conjunction with graph theoretical analysis, was used to construct functional connectivity matrices and to extract the attribute features of the small-world networks in insomnia. We examined the alterations in global and local small-world network properties of the distributed brain regions that are predominantly implicated in the frontostriatal network between 30 healthy subjects with insomnia symptoms (IS) and 62 healthy subjects without insomnia symptoms (NIS). Correlations between the small-world properties and clinical measurements were also generated to identify the differences between the two groups. Both the IS group and the NIS group exhibited a small-worldness topology. Meanwhile, the global topological properties didn't show significant difference between the two groups. By contrast, participants in the IS group showed decreased regional degree and efficiency in the left inferior frontal gyrus (IFG) compared with subjects in the NIS group. More specifically, significantly decreased nodal efficiency in the IFG was found to be negatively associated with insomnia scores, whereas the abnormal changes in nodal betweenness centrality of the right putamen were positively correlated with insomnia scores. Our findings suggested that the aberrant topology of the salience network and frontostriatal connectivity is linked to insomnia, which can serve as an important biomarker for insomnia.

## Introduction

Insomnia is the most prevalent sleep disorder and is characterized by difficulties in falling asleep at bedtime, frequent awakenings in the middle of the night, and waking up too early in the morning (Morin and Benca, 2012; Cheung et al., 2013; Riedner et al., 2015). It has a high prevalence, in which 30~50% of the general population suffers from insomnia symptoms, with an increased incidence rate with older age, and up to 10% of the population meets the clinical criteria for insomnia disorder (Ohayon, 2002; Moore, 2012; Morin and Benca, 2012; Kronholm et al., 2015). More importantly, insomnia has been recognized to be a substantially increased potential trigger for the development of other mental illnesses, such as depression. Previous work has demonstrated that insomnia individuals are approximately 10 times more likely to develop depression as compared with good sleepers, and approximately 90% of patients with depression also suffer from insomnia (Taylor et al., 2005; Kaneita et al., 2006; Ohayon and Hong, 2006; Benca and Peterson, 2008; Wulff et al., 2010; Mayer et al., 2011). In particular, insomnia is strongly associated with feeling fatigued in the performance of academic functioning, a reduced working memory performance, feeling anxiety in complex tasks, the overuse of drugs and alcohol, the presence of suicidal ideation, and a poor life quality in adults, children and adolescents (Short et al., 2013; Kronholm et al., 2015). Accordingly, insomnia is also associated with a marked increase in health-care consumption, poor occupational performance, and high costs for society (Kucharczyk et al., 2012; Moore, 2012; Lian et al., 2015). Despite the huge socio-economic impact caused by insomnia, the neurobiological mechanisms that underlie insomnia remain unclear, thereby hampering the development of effective treatments.

Recently, numerous structural and functional neuroimaging studies have examined widespread dysfunctions in the fronto-striato-limbic-thalamic regions in insomnia (Drummond et al., 2004; Spiegelhalder et al., 2013, 2015; Riemann et al., 2015). For example, using high-resolution 3D T1-weighted magnetic resonance imaging (MRI), Altena et al. discovered that the gray matter volume (GMV) in the left orbitofrontal cortex (OFC) was decreased in 24 insomnia patients, and the GMV of the OFC was strongly positively associated with insomnia scores (Altena et al., 2010). Additionally, Stoffers et al. used the whole-brain voxel-based morphometry (VBM) to access the gray matter density of 65 healthy subjects with insomnia symptoms, in which a negative relationship between the gray matter density in the left OFC and earlier morning awakening was identified (Stoffers et al., 2012). Joo et al. also found a reduced GMV in multiple brain regions including the bilateral frontal lobes based on measurements from 27 patients with insomnia (Joo et al., 2013). In particular, a further VBM study revealed GMV reductions in the bilateral OFC and the adjacent bilateral inferior frontal gyrus (IFG) pars orbitalis were correlated with increased sleep fragmentation, which was objectively quantified via actigraphy in 141 older community-dwelling adults (Lim et al., 2015). Further, the GMV reduction of frontal areas was also identified, which was related to decreased integrity of white matter tracts in the anterior internal capsule, as demonstrated by a recent diffusion tensor imaging study in 24 insomnia patients (Spiegelhalder et al., 2014).

In addition to the changes in the GMV, using task-based functional MRI (fMRI), Altena et al. found a hypoactivation in the inferior prefrontal cortex during daytime task performance in 21 patients with insomnia (Altena et al., 2008). Interestingly, findings from Drummond et al.'s analysis suggested that decreased activations in the frontal and thalamic brain regions were present during the performance of working memory tasks for 25 individuals with insomnia, which demonstrated dysfunctions in the fronto-striatal networks for insomnia (Drummond et al., 2013). In addition, based on a seed-based functional connectivity analysis method, Huang et al. identified a decreased connectivity between the frontal regions and the limbic areas in 10 medication-naive insomnia patients, which illustrated impairments in the fronto-limbic systems (Huang et al., 2012). Further, Nie et al. found that compared with good sleepers, patients with insomnia exhibited a significantly decreased functional connectivity between the medial prefrontal cortex [a core region of the default-mode network (DMN)] and the right medial temporal lobe (Nie et al., 2015). Li et al. used the resting-state fMRI (rsfMRI) to investigate 55 insomnia patients, which unveiled decreased amplitude of low-frequency fluctuation (ALFF) in the left OFC/IFG and the right middle frontal gyrus in insomnia (Li et al., 2016). An additional ALFF study performed by Zhou et al. revealed that significantly decreased ALFF regions were mainly detected in the prefrontal cortex and DMN sub-regions, which indicated that abnormal sensory input and intrinsic information processing could be a hyperarousal state in insomnia (Zhou et al., 2016). More recently, Stoffers et al. used an executive task to evaluate fronto-striatal functioning in 24 disturbed sleep subjects because the fronto-striatal networks play an important role in sleep, arousal regulation and executive function, and their findings revealed that a reduced recruitment of the left caudate nucleus was involved in the pathophysiology of insomnia (Stoffers et al., 2014).

Taken together, the important neuroimaging findings described above have indicated that dysfunctions in the frontal-based circuits and striatal-based systems played an essential role in identification of the neurobiological mechanism of insomnia. To date, most previous work on the analysis of disrupted networks in insomnia has predominately centered on the seed-based method. For this method, the seed was first defined according to the previous knowledge regarding the anatomical information or task-based activation, and the intrinsic functional connectivity was generated based on the correlation between this seed and all other brain areas. However, the seed-based connectivity measurement is unable to investigate the disconnectivity without a prediction of *a priori* regions of interest (ROIs). This is particularly challenging in the identification of accurate seeds/ROIs within the large, heterogeneous, and insomnia-related regions, such as the frontal cortex and the striatum. In contrast, graph theoretical analysis, as an alternative data-driven approach, provides the basis for improving understanding of the topological properties of brain networks independent of *a priori* seeds. It delineated the brain as a large-scale network that consists of nodes (brain regions) and edges (functional connections between pairs of nodes) (Bullmore and Sporns, 2009). The high-level topological properties may be sensitive to influences that cannot be detected by seed-based methods. Importantly, graph theoretical analysis can be adopted to investigate the functional abnormalities in insomnia at both the global and nodal levels. For example, at the global level, Watts and Strogatz demonstrated that the small-world network may be characterized by a higher clustering coefficient (the ratio of the number of existing interconnections of a node with its node neighbors to the maximum of all possible connections) and a similar shorter path length (the average number of minimum links that are required to travel between two nodes) compared with a random network (Watts and Strogatz, 1998). By contrast, at the nodal level, the node degree measured the connectedness of an isolated node with all other nodes, which may identify highly connected nodes that may play key roles in information integration in a network.

More importantly, evidences from neuroimaging studies have demonstrated that the human brain has a small-world network topology that may be disrupted in psychiatric and neurological disorders, such as depression (Meng et al., 2014; Long et al., 2015), schizophrenia (Liu F et al., 2016), Alzheimer's disease (Wang et al., 2013), epilepsy (Zhang Z et al., 2011), obsessive-compulsive disorder (Zhang T et al., 2011), autism (Itahashi et al., 2014), attention-deficit/hyperactivity disorder (Wang L et al., 2009), multiple sclerosis (He et al., 2009), post-traumatic stress disorder (Long et al., 2013), and spinal cord injury (De Vico Fallani et al., 2007). In this study, we determined to investigate whether the functional brain networks in insomnia also have small-world properties. We examined the topological properties of the whole brain networks between healthy subjects with insomnia symptoms (IS) and healthy subjects without insomnia symptoms (NIS). In particular, we explored the functional brain networks and the small-world network properties of insomnia generated by using rsfMRI data and graph theoretical analysis. Specifically, we hypothesized that (1) both the IS group and the NIS group should exhibit small-world properties; (2) the IS group can exhibit a disrupted functional topological organization and show altered local network properties of distributed regions predominantly within the frontal and striatal regions compared with the NIS group; and (3) an altered topology of the frontal and striatal regions should be involved in the salience network and frontostriatal circuits for the IS group.

To test these hypotheses, rsfMRI was used to explore the intrinsic brain activity in 30 healthy subjects with insomnia symptoms and 62 healthy subjects without insomnia symptoms using spontaneous low-frequency (0.01~0.1 Hz) fluctuations in the blood oxygen level-dependent signal during the resting state (Biswal et al., 1995, 1997). In addition, graph theoretical analysis was also used to explore the topological organization of the functional connectivity networks. The global and nodal network properties were examined and compared between the IS and NIS groups, and the clinical relevance of the aberrant brain network topologies was also evaluated. Certainly, this pilot work will pave a new avenue for an improved understanding of the neural mechanism that underlies insomnia.

## Materials and methods

### Participants

One hundred and two right-handed, healthy subjects (53 females, 49 males; mean age ± SD: 36.83 ± 12.36 years; years of education: 15.00 ± 3.51 years) were recruited to participate in the study. The subjects were screened by two psychiatrists (LRT and CLT) by using the Non-Patient Structured Clinical Interview for the Diagnostic and Statistical Manual of Mental Disorders (DSM-IV) (SCID). Participants with a current or lifetime history of psychiatric or neurological illnesses, such as depression, anxiety disorders, schizophrenia, epilepsy, chronic pain or traumatic brain damage, were excluded from this study. All participants had no histories of alcohol, substance abuse or dependence. Written informed consents were collected for all participants prior to the experimental tests. All clinical tests and protocol for the present work were approved by the Medical Ethics Committee of Beijing Anding Hospital with the Capital Medical University, the Imaging Center for Brain Research with the Beijing Normal University, and the Biomedical Ethics Board with the Faculty of Health Sciences at the University of Macau (Macao SAR, China) in accordance with the approved guidelines.

### Clinical measurements

The 17-item Hamilton Depression Rating Scale (HAMD-17) and the Hamilton Anxiety Rating Scale (HAMA) were used to measure the severity of depression and anxiety (Hamilton, 1967). Insomnia symptoms were assessed according to the total score of the three-item sleep subscale on the HAMD-17. Three items which were scored 0, 1, or 2, were applied to evaluate the difficulty in initiating sleep (DIS, early insomnia), the difficulty in maintaining sleep (DMS, middle insomnia), and the early morning awakening (EMA, late insomnia), respectively. In particular, DIS is defined as a difficulty in falling asleep, DMS is denoted as a difficulty in maintaining sleep that leads to frequent awakenings or a difficulty in returning to sleep after awakenings, and EMA indicates early morning awakening with an inability to return to sleep. The participants who had a total score equal to or greater than 1 were considered to possess insomnia symptoms. It is noted here that the HAMD-17 sleep items were adopted to evaluate the three stages of insomnia instead of the Pittsburgh Insomnia Rating Scale that involved not only insomnia but also the subjective sleep quality, the sleep latency and duration, the use of sleep medication, and daytime dysfunction account (Trivedi et al., 2013). However, previous work showed that the sleep items measured by the HAMD were better correlated with sleep diaries (Manber et al., 2005). More notably, we generated the adjusted HAMD scores and adjusted HAMA scores without using the insomnia-related items to reduce the potential influence of insomnia via the HAMD and HAMA on our diagnostic results.

### Data acquisition

Data acquisition was performed on a 3.0-Tesla MRI scanner (Siemens Medical Solutions, Erlangen, Germany) in the National Key Laboratory for Cognitive Neuroscience and Learning, Beijing Normal University. In particular, no medicine were taken for all participates before the MRI recordings. The rsfMRI data were collected using an echo-planar imaging (EPI) sequence with the following scanning parameters: TR = 2 s, TE = 30 ms, flip angle = 90°, FOV = 220 × 220 mm, in-plane matrix size = 64 × 64, number of slices = 33, slice thickness = 3.5 mm, and inter-slice gap = 0.6 mm. All participants were instructed to completely relax without thinking of special things, simply rest quietly with their eyes closed, remain still, and remain awake during the data recording. For each subject, the overall resting-state data acquisition lasted 8 min, resulting in 240 volumes. No subject dropped out of the experiment during the scanning.

### Data preprocessing

All single-subject resting-state data preprocessing steps were performed using the Data Processing Assistant for Rest-State fMRI (DPARSF) toolbox (Chao-Gan and Yu-Feng, 2010). The first 10 volumes were discarded from each participant in favor of steady-state longitudinal magnetization. The remaining resting-state fMRI data were first slice-timing corrected and then realigned to the first of the remaining volumes for head-motion correction. Following these corrections, the functional images were spatially normalized into the standard Montreal Neurological Institute (MNI) space with a resliced resolution at 3 × 3 × 3 mm^3^. No spatial smoothing was applied to avert the introduction of an artificial local spatial correlation according to previous studies (Salvador et al., 2005; Achard et al., 2006; Achard and Bullmore, 2007; Wang J et al., 2009; Liao et al., 2010b). To remove the several spurious sources of variance, the six head motion parameters and the averaged signals from the white matter signals and cerebrospinal fluid signals were regressed out using the CompCor method (Behzadi et al., 2007). No global signal was regressed out to avoid yielding spurious negative correlations (Murphy et al., 2009). In addition, the resulting time series was detrended to remove the linear trend and then temporally bandpass filtered (0.01~0.1 Hz) to reduce the effects of low-frequency drifts and high-frequency physiological noises. Furthermore, a “scrubbing” method together with a frame-wise displacements (FD) threshold of 0.5 mm were implicated to remove the contaminated time points and reduce the impact of motion artifacts induced by systematic biases on the time series (Power et al., 2012). Specifically, at each time point *t*, the estimation of the motion FD was computed from three head translations (*d*_*tx*_, *d*_*ty*_, *d*_*tz*_) and three head rotations (*d*_*tα*_, *d*_*tβ*_, *d*_*tγ*_) using the formula: *FD*_*t*_ = |Δ*fd*_*tx*_|+|Δ*fd*_*ty*_|+|Δ*fd*_*tz*_|+|Δ*fd*_*tα*_|+|Δ*fd*_*tβ*_|+|Δ*fd*_*tγ*_|, in which the Δ*fd*_*tx*_ = *fd*_(*t*−1)*x*_−*fd*_*tx*_, and the similarity calculation procedure was performed for the other rigid body parameters (*d*_*ty*_, *d*_*tz*_, *d*_*tα*_, *d*_*tβ*_, *d*_*tγ*_). In particular, the angle rotational displacements were generated and transformed from degrees to millimeters by applying a radius of *r* = 50 mm, which is approximately the mean distance between the center of the MNI space and the cortex. Recordings at time points when *FD*_*t*_ > 0.3 mm were considered to be potentially contaminated with motion artifacts and should be eliminated from the time series for further analysis (Power et al., 2012). The root mean squared variance of the voxels was also calculated to measure the degree to which the intensity of a brain image has changed compared with previous time points (Power et al., 2012). Finally, no data exceeded ± 2 mm or ± 2° For their translational or rotational parameters, respectively. However, the data from 10 subjects were discarded from further analysis because their data were cut off by more than 50% of their original volumes after scrubbing procedures.

### Network construction

To generate a brain network, it is essential to define the nodes and edges of the networks. To determine the nodes and edges of brain functional connectivity networks, the following procedure was applied as methods previously described (Gong et al., 2009; Zhang J et al., 2011).

#### Network node definition

To define the nodes of brain networks, the automated anatomical labeling (AAL) atlas (Tzourio-Mazoyer et al., 2002) was utilized to parcellate the whole brain into 90 anatomical ROIs, with 45 ROIs per hemisphere, and each ROI represented a node of the network. Detailed information on the ROIs is given in Table S1 as the supplementary material.

#### Network edge definition

To define the edges of the brain networks, a representative time series of each ROI from each subject was generated by averaging the time series of all voxels within the ROI. A 90 by 90 temporal correlation matrix was then constructed for each subject by calculating the Pearson's correlation coefficients between the time series from all pairs of ROIs. Further, a Fisher's r-to-z transformation was applied to the correlation matrices, and the individual correlation matrices were thresholded into binarized matrices with a sparsity value *T* (defined as the ratio of the total number of edges in a network to the maximum possible number of edges to ensure that the networks from two comparison groups have the same number of edges or wiring costs). When the Pearson's correlation coefficient was greater than *T*, the corresponding edge was considered as existence in brain networks.

### Network analysis

#### Threshold selection

Instead of selecting a single threshold *T*, we applied a range of sparsity values from 0.11 to 0.30 with an interval of 0.01 according to the criteria suggested in previous studies (Zhang J et al., 2011). Specifically, (1) the minimum sparsity was selected to ensure that the mean degree across all nodes of each thresholded network was greater than 2 × log(*N*), where *N* = 90 is the number of nodes, and (2) the maximum sparsity was selected to ensure that the small-worldness scalar of each thresholded network was greater than 1.1 for all participants.

#### Small-world network measurements

Graph theoretical analysis was carried out using the graph theoretical network analysis (GRETNA) toolbox (Version 1.2, https://www.nitrc.org/projects/gretna/) to measure the network topological properties and examine the functional connectivity organization (Rubinov and Sporns, 2010; Wang et al., 2015). The small-world network properties, including the clustering coefficient of the network, *C*_*p*_, and the path length, *L*_*p*_, were also generated by using the method initially proposed by Watts and Strogatz (Watts and Strogatz, 1998).

To quantify the small-world characteristics, 100 random networks were constructed by using a Markov-chain algorithm at each sparsity threshold for each individual network with the same degree distribution as the functional connectivity networks (Liao et al., 2010a). Typically, we scaled *C*_*p*_ and *L*_*p*_ of the examined functional connectivity networks to the averaged *C*_*random*_ and *L*_*random*_ from all 100 random networks (i.e., the normalized clustering coefficient, lambda $\;\gamma {=}^{\;C_{p}}{/}_{C_{random}}$, and the normalized characteristic path length, gamma $\;\lambda {=}^{\;L_{p}}{/}_{L_{random}}$). These two parameters can also be integrated into a quantitative measurement, sigma $\sigma {=}^{\lambda }{/}_{\gamma }$, which is denoted as small-worldness. A real network is recognized to own small-world network properties if it satisfies the following conditions: (1) γ > 1 and λ ≈ 1 (Watts and Strogatz, 1998); (2) σ > 1 (namely, the small-world network has a higher clustering coefficient and a similar path length compared with the random network) (Humphries et al., 2006; Liu et al., 2008). The descriptions of the measurements were provided in Table S2.

#### Regional nodal measurements

The regional nodal properties may be characterized by a number of key measurements, including the nodal degree *Deg*_*i*_, the nodal efficiency *E*_*nodal*_, and the nodal betweenness *BC*_*i*_ (Table S2). The nodal degree is defined as the number of nodes in a subgraph *G*_*i*_, which is the graph that includes the nodes that are direct neighbors of node *i*. The nodal degree evaluates the extent to which the node is connected to the remaining nodes in a network. The nodal efficiency *E*_*nodal*_ is the inverse of the harmonic mean of the length between node *i* and all other nodes in the network to address the disconnected graphs, non-sparse graphs, or both factors, which measures the level of information propagation of a node with all other nodes in the network. The nodal betweenness *BC*_*i*_ is denoted as the fraction of all the shortest paths in the network that pass through node *i*, which estimates the influence of a node over the information flow with the remaining nodes in a network.

#### Efficiency of small-world networks

There are two indicators to define the network efficiency: global efficiency *E*_*glo*_ and local efficiency *E*_*loc*_ (Achard and Bullmore, 2007) (Table S2). We first calculated the six global network parameters, *C*_*p*_, *L*_*p*_, λ, γ, *E*_*glo*_, and *E*_*loc*_, and three regional nodal properties, *Deg*_*i*_, *E*_*nodal*_, and *BC*_*i*_. Then, we generated a summarized scalar for the topological characterization of brain functional networks by computing the area under the curve (AUC) based on each network parameter (Zhang J et al., 2011). Detailed descriptions on the definition and calculation of the network properties were provided in the supplementary materials.

### Statistical analysis

#### Differences in the network properties

In this study, the remaining 92 subjects were divided into two groups: the IS group (*n* = 30), who had insomnia scores greater than or equivalent to 1, and the NIS group (*n* = 62), who had insomnia scores equivalent to 0. Subjects from the two groups were matched in age, gender, and educational level, and their demographic information was provided in Table 1. To compare the global network topological properties (*C*_*p*_, *L*_*p*_, *E*_*glo*_, *E*_*loc*_, λ, γ, and σ) between the IS and NIS groups, a series of two-sample *t*-tests (*p* < 0.05, uncorrected) were performed for each property across the preselected sparsity thresholds, 0.11 ≤ *T* ≤ 0.30, with an interval of 0.01. To show the distinctions in the regional nodal properties (*Deg*_*i*_, *E*_*nodal*_ and *BC*_*i*_) between the two groups, nonparametric permutation tests (5,000 iterations, *p* < 0.05, uncorrected, controlling for age, gender, educational level, adjusted HAMA score, and adjusted HAMD score) were also performed on the AUC generated from each nodal property (Bullmore et al., 1999). Briefly, we first computed the actual between-group differences in the AUC of each network metric. Then, we put this difference into a null permutation distribution of the differences to recalculate by chance by randomly assigning the values of each subject to two randomized groups with the same sizes as the IS and NIS groups. This procedure was repeated for 5,000 permutations. Before the permutation tests, a multiple regression analysis was conducted with age, gender, educational level, adjusted HAMA score, and adjusted HAMD score as the dependent variables and the AUC of each network metric as the independent variable as well. A *p* < 0.05 for multiple comparisons was considered significant. Furthermore, Pearson's correlation analysis was utilized to inspect the relationship in the AUC of the nodal network properties between the insula and other brain regions that showed significant difference between the IS and NIS groups.

**Table 1:** **Demographic and clinical data**.

**Measure**	**IS (*n* = 30)**	**NIS (*n* = 62)**	***t***	***p*****-value**
Age (years)	38.00 ± 11.85	37.47 ± 11.95	0.201	0.841^*^
Gender (male/female)	15/15	26/36	0.532	0.466^#^
Education level (years)	14.07 ± 3.34	15.42 ± 2.95	−1.889	0.065^*^
HAMD score	2.93 ± 1.46	0.19 ± 0.51	9.987	<0.001^*^
Adjusted HAMD score	1.23 ± 1.31	0.19 ± 0.51	4.214	<0.001^*^
HAMA score	3.20 ± 2.12	0.32 ± 0.79	7.187	<0.001^*^
Adjusted HAMA score	1.97 ± 2.00	0.32 ± 0.79	4.327	<0.001^*^
Insomnia score	1.70 ± 0.92	0.00 ± 0.00	10.172	<0.001^*^

*Data are presented as mean ± SD*.

* *indicates p values for two-sample two-tailed t-tests*;

# *indicates p values for χ^2^-test. Adjusted HAMD score means HAMD scores after omission of sleep questions. Adjusted HAMA score means HAMA scores after omission of sleep questions. IS, healthy subjects with insomnia symptoms; NIS, healthy subjects without insomnia symptoms; SD, standard deviation; HAMD, Hamilton Depression Rating Scale; HAMA, Hamilton Anxiety Rating Scale*.

#### Correlations between the network properties and insomnia scores

Pearson's correlation analysis was performed to assess the relationship between the network properties (both global and nodal) and insomnia scores in the IS and NIS groups with age, gender, educational level, adjusted HAMA score, and adjusted HAMD score as covariates (independent variables: AUC of each network property; dependent variables: insomnia scores of all subjects). A value of 1/number of nodes = 1/90 = 0.011 was considered as a significant threshold for false-positive correction for all analyses.

## Results

### Demographic and clinical comparisons between the IS and NIS groups

Table 1 presented the information on the demographics and clinical variables for the two groups. Both the original HAMD scores and the original HAMA scores in the IS group were higher than that in the NIS group (*p* < 0.001; Table 1). Additionally, the adjusted HAMD scores and the adjusted HAMA scores of the IS group were also higher as compared to that from the NIS group (*p* < 0.001; Table 1).

### Differences in the global network properties between the *Two* groups

**Figure 1:**
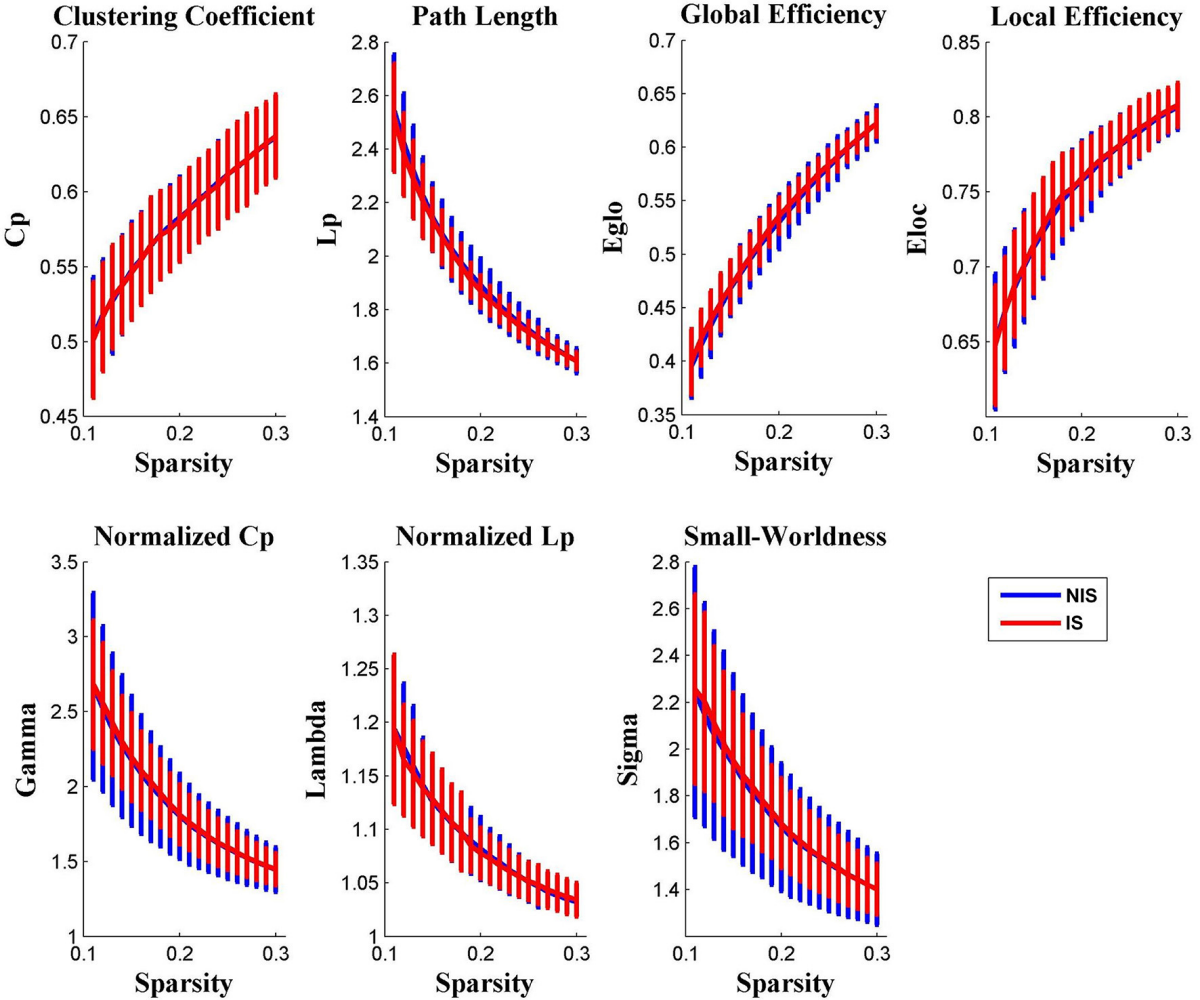
**Group comparison of global network topological properties (*C*_*p*_, *L*_*p*_, *E*_*glo*_, *E*_*loc*_, λ, γ, and σ) between the IS and NIS group (two-sample *t*-tests, *p* < 0.05, uncorrected)**. The small-worldness suggests a small-world topology for functional brain networks of both the IS and NIS group. The error bar represents the standard deviation (SD). Cp, clustering coefficient; Lp, path length; E_glo_, global efficiency; E_loc_, local efficiency. IS, healthy participants with insomnia symptoms, NIS, healthy participants without insomnia symptoms.

It was discovered from Figure 1 that the functional brain networks of both the IS and NIS groups exhibited small-world properties, in which the small-worldness index of sigma was greater than 1.1 for all threshold values ranged from 0.11 to 0.30. In addition, as displayed in Figure 1, all other global network properties didn't show significantly difference between the two groups across the entire scopes of sparsity for the correlation coefficients.

### Alterations in regional nodal network properties of salience networks

**Figure 2:**
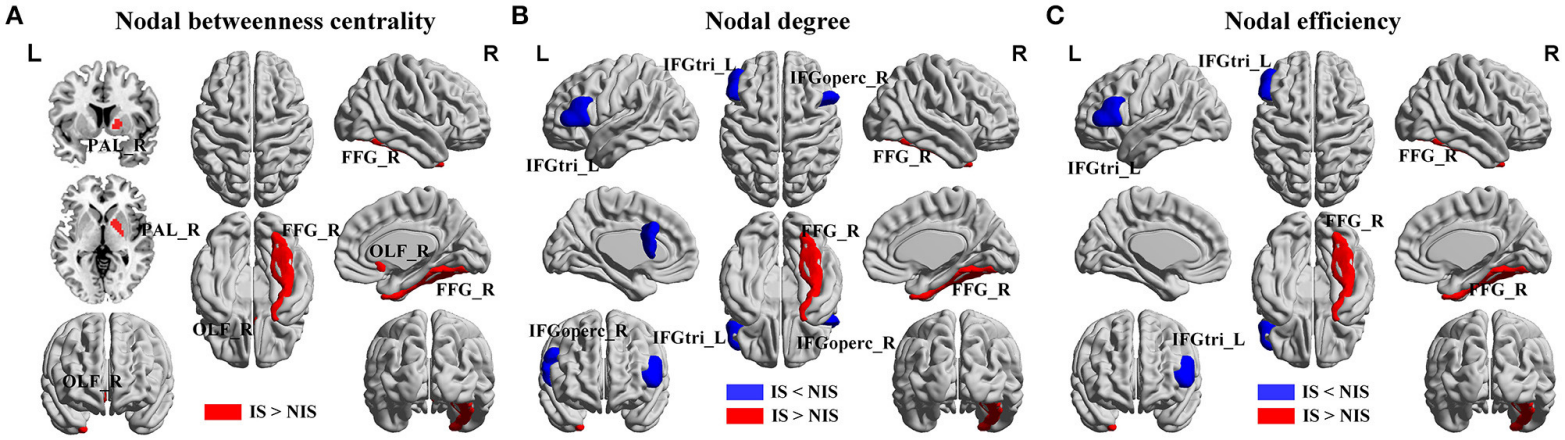
**Brain regions showing altered local network properties in IS group as compared with NIS group**. The aberrant **(A)** nodal betweenness centrality, **(B)** nodal degree, and **(C)** nodal efficiency were observed in IS group. Group comparisons were based on permutation tests (5,000 permutations, *p* < 0.05, controlling for the age, gender, educational level, adjusted HAMA score, and adjusted HAMD score). Colored brain areas indicate the significantly altered local network properties in IS group. The blue and red regions represent significantly decreased and increased nodal network properties in IS group as compared with NIS group, respectively. The more detailed information were presented in Table 2. IS, healthy participants with insomnia symptoms; NIS, healthy participants without insomnia symptoms; L, left; R, right; FFG, fusiform gyrus; PAL, pallidum; OLF, olfactory cortex; IFGoperc, inferior frontal gyrus pars opercularis; IFGtri, inferior frontal gyrus pars triangularis. Figures were visualized using the BrainNet Viewer software (https://www.nitrc.org/projects/bnv/).

Alterations in the nodal betweenness centrality, nodal degree and nodal efficiency were identified in several brain regions of the subjects in the IS group (*p* < 0.05, uncorrected) (Figure 2 and Table 2). In particular, compared with the NIS group, the IS group exhibited increased nodal betweenness centrality in the right fusiform (FFG), right pallidum (PAL), and right olfactory cortex (Figure 2A and Table 2). Meanwhile, decreased nodal degree in the left IFG including the right inferior frontal gyrus pars opercularis (IFGoperc) and the left inferior frontal gyrus pars triangularis (IFGtri), and increased nodal degree in the right FFG were identified in the IS group compared with that from the NIS group (Figure 2B and Table 2). In addition, the IS group also showed decreased nodal efficiency in the left IFGtri and increased nodal efficiency in the right FFG compared with the NIS group (Figure 2C and Table 2). Intriguingly, regarding the regional nodal degree, we discovered that for the IS group, the right IFGoperc was positively correlated with the right insula of the brain (*r* = 0.274, *p* = 0.008). Further, it was also observed from Table 2 that insomnia scores were associated with the aberrant nodal network topology of frontostriatal connectivity.

**Table 2:** **Brain regions showing abnormal nodal network properties in IS as compared with NIS**.

**Brain regions**	**Side**	**Nodal network property**	**IS**	**NIS**	***p-*****value**
**IS<NIS**					
IFGoperc	R	AUC of Deg	2.45 ± 1.07	2.92 ± 1.26	0.017
IFGtri	L	AUC of Deg	2.03 ± 0.65	2.68 ± 1.25	0.016
IFGtri	L	AUC of E_nodal_	0.09 ± 0.01	0.10 ± 0.01	0.025
**IS > NIS**					
FFG	R	AUC of BC	14.28 ± 9.73	10.82 ± 8.65	0.043
OLF	R	AUC of BC	5.68 ± 7.74	6.62 ± 4.02	0.049
PAL	R	AUC of BC	4.55 ± 6.58	2.49 ± 3.85	0.031
FFG	R	AUC of Deg	5.33 ± 1.38	4.68 ± 2.68	0.043
FFG	R	AUC of E_nodal_	0.12 ± 0.01	0.11 ± 0.01	0.041

*Group comparisons: permutation tests (5,000 permutations, p < 0.05, controlling for the age, gender, educational level, adjusted HAMA score, and adjusted HAMD score). The AUC of the nodal network properties was calculated over the range of 0.11 ≤ T ≤ 0.30 with an interval of 0.01. Data are reported as mean ± SD. IFGoperc, inferior frontal gyrus pars opercularis; IFGtri, inferior frontal gyrus pars triangularis; FFG, fusiform gyrus; PAL, pallidum; OLF, olfactory cortex; IS, healthy participants with insomnia symptoms, NIS, healthy participants without insomnia symptoms. R, right; L, left; AUC, area under the curve; BC, nodal betweenness centrality; Deg, nodal degree; E_nodal_, nodal efficiency*.

**Figure 3:**
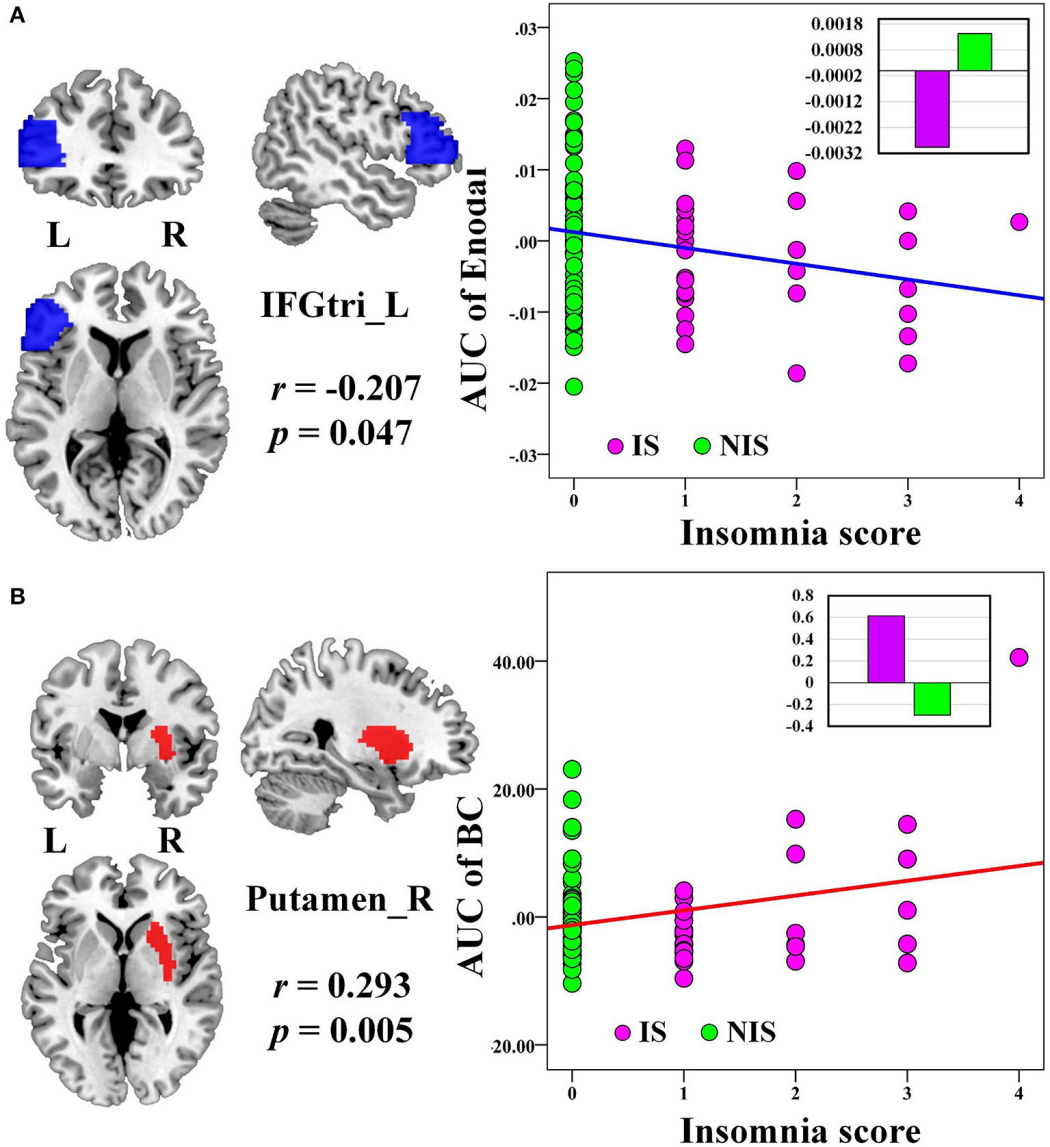
**The Pearson correlation between the AUC of the nodal network properties with insomnia scores in all the subjects (*p* < 0.05, uncorrected, controlling for the age, gender, educational level, adjusted HAMA score, and adjusted HAMD score)**. The AUC of each nodal topology was calculated over the range of 0.11 ≤ *T* ≤ 0.30 with an interval of 0.01. The red color reveals the positive correlation while the blue color represents the negative correlation. The violet circles stand for the IS group while the green circles indicate the NIS group. The bar plots stand for the mean value of each AUC of nodal network properties in corresponding brain regions for IS and NIS groups. The more detailed information were presented in Table 3. L, left; R, right; AUC, area under the curve; BC, nodal betweenness centrality; E_nodal_, nodal efficiency; IFGtri, inferior frontal gyrus pars triangularis; IS, healthy participants with insomnia symptoms; NIS, healthy participants without insomnia symptoms.

No significant correlation was identified between the global network metrics (*C*_*p*_, *L*_*p*_, *E*_*glo*_, *E*_*loc*_, λ, γ, and σ) and the insomnia scores across all subjects. However, the AUC of the nodal efficiency in left IFGtri was negatively correlated with the insomnia scores (*p* < 0.05, uncorrected) (Figure 3A and Table 3). What's more, the AUC of the nodal betweenness centrality of the right putamen (*p* < 0.011) (Figure 3B and Table 3), the right fusiform gyrus (FFG) (*p* < 0.05) (Table 3), and the right middle frontal gyrus (MFG) (*p* < 0.05) (Table 3) were found to be positively associated with the insomnia scores. In particular, the altered nodal network topology in the IFGtri and putamen was identified to be related with the abnormal frontostriatal connectivity in insomnia.

**Table 3:** **Significant correlations between nodal network properties and insomnia scores across all subjects**.

**Node**	**Side**	**Nodal network property**	***r-*****value**	***p*****-value**
**DECREASED ASSOCIATION BETWEEN NODAL NETWORK PROPERTY WITH INSOMNIA SCORE**				
Inferior frontal gyrus, triangular part	L	AUC of E_nodal_	−0.207	0.047
**INCREASED ASSOCIATION BETWEEN NODAL NETWORK PROPERTY WITH INSOMNIA SCORE**				
Putamen	R	AUC of BC	0.293	0.005^*^
Fusiform gyrus	R	AUC of BC	0.236	0.024
Middle frontal gyrus	R	AUC of BC	0.220	0.035

*Pearson correlation analyses were corrected controlling for the age, gender, educational level, adjusted HAMA score, and adjusted HAMD score. The AUC of the nodal topology was calculated over the range of 0.11 ≤ T ≤ 0.30 with an interval of 0.01. R, right; L, left; AUC, area under the curve; BC, nodal betweenness centrality; E_nodal_, nodal efficiency*.

* *Reported results are significant for p < 1/90 based on false positive correlation for multiple comparisons*.

## Discussion

In the current study, we examined how the topological organization of the intrinsic brain networks is associated with insomnia in 30 healthy individuals with insomnia and 62 healthy individuals without insomnia. The comparison of the measurement of small-worldness indicated that the differences in the small-world properties of the brain activation networks between the IS and NIS groups were not significant. However, compared with the NIS group, the IS group exhibited decreased nodal degree in the IFG including the right IFGoperc and the left IFGtri, and decreased nodal efficiency in the left IFGtri. Specifically, it is noted that the nodal efficiency of the left IFGtri was significantly negatively correlated with insomnia scores, whereas the nodal betweenness centrality in the right putamen was positively correlated with insomnia scores. In particular, the insomnia score was found to be selectively associated with the aberrant nodal network properties in the left IFGtri and right putamen. By contrast, the regional nodal degree in right IFGoperc was positively associated with the right insula, a key component of the salience network. The identified neural markers demonstrated that the insomnia without any underlying psychiatric comorbidity can be characterized by the altered network topology of the IFG of the salience network. These novel findings also provided evidence that disrupted topological organizations of the frontostriatal connectivity and salience network were associated with insomnia, suggesting that these networks contributed to the neural circuitry that underlies insomnia. In addition, the identified neural markers were independent of the age, gender or educational level, which are controlled for in the group comparison. Further, previous work showed that insomnia has close link with depression and/or anxiety disorders (Ford and Kamerow, 1989; Gillin, 1998; Riemann and Voderholzer, 2003; Staner, 2010). However, in the preset study, the neuroimaging results were also controlled for the adjusted HAMA scores and adjusted HAMD scores to eliminate the influence of depression and/or anxiety disorders, indicating that insomnia rather than anxiety disorders or depression is associated with an altered topology of the frontostriatal connectivity. Consequently, our novel findings suggested that altered nodal network properties in areas of the salience network and frontostriatal connectivity are only linked with insomnia.

Consistent with our hypothesis, we discovered that the IS group showed a disrupted functional topological organization compared with the NIS group. A decreased nodal degree and nodal efficiency of the distributed brain regions were identified predominantly within the left IFGtri in insomnia, whereas decreased nodal degree was also revealed in the right IFGoperc for the NIS group. In addition, the nodal efficiency of the left IFGtri was negatively associated with the insomnia score. As such, our results exhibited decreased nodal degree and nodal efficiency and revealed a negative association between the nodal network topology in the IFG and insomnia, indicating that more severe insomnia was associated with a more disrupted IFG and a weaker IFG hubness for information integration.

The prefrontal cortex has demonstrated its important roles in alertness, attention, and higher-order cognitive processes for insomnia (Thomas et al., 2000; Spiegelhalder et al., 2015). Recently, Li et al. demonstrated reduced ALFF values in the left IFG and right MFG in primary insomnia (PI) patients. In addition, they also revealed a negative correlation between the duration of PI and ALFF values in the left IFG (Li et al., 2016). Using task-based fMRI, Martin et al. reported that poor quality sleepers showed smaller activations in the right IFG, right MFG, and bilateral insula compared with good quality sleepers during an immediate and impulsive monetary decision task (Martin et al., 2015). By using the superior parietal lobe (SPL) as a seed, Li et al. illustrated that insomnia patients exhibited increased connectivity between the SPL and the right IFGtri, right insula lobe, right ventral anterior cingulate cortex (ACC), left ventral posterior cingulate cortex, and right splenium of the corpus callosum, which represent brain regions critical for spatial and verbal working memory (Li et al., 2014). Moreover, Altena et al. discovered that insomnia patients showed a hypoactivation in the IFG during performance in a category and letter fluency task, which recovered after sleep therapy. These findings suggested that insomnia disrupted cognitive functions (Altena et al., 2008). In Spira's study, the subjects with a sleep duration of less than 7 h exhibited increased rates of subsequent gray matter atrophy in the IFG and MFG compared with those with more than 7 h of sleep (Spira et al., 2015). Liu et al. found that a sleep deprivation group showed increased resting-state functional connectivity between the left cerebellum posterior lobe and the right IFG compared with the good sleepers group (Liu et al., 2015). Further, Shao et al. demonstrated that following total sleep deprivation, subjects with insomnia exhibited a significant decrease in the functional connectivity between the amygdala and several executive control regions, including the right IFG, right dorsal ACC, and left dorsolateral prefrontal cortex, indicating disturbances in the IFG-amygdala circuits in sleep (Shao et al., 2014). Using PET recordings, Wu et al. found a significant reduction in glucose metabolism in the IFG, MFG, medial frontal gyrus, and superior frontal gyrus after sleep deprivation (Wu et al., 2006). Consequently, the identified neural markers for the present work are consistent with those previous findings. Decreased nodal network topology of the IFG in this study indicated its weakened role in coordinating whole-brain networks, which sheds light on the understanding of the pathophysiology of insomnia.

In addition, a positive correlation was identified between the nodal degree of the IFGoperc and the insula. Notably, the anterior insula is adjacent to the IFG, which may be jointly referred to as the insula/IFG cortex. The anterior insula associated with IFG is the key hub of the salience network, which is interconnected with diverse cortical regions including the amygdala, medial temporal lobe, and basal ganglia (Kelly et al., 2012; Tahmasian et al., 2016). As a result, it is not surprising that the insula plays key roles in saliency detection, decision-making, motor/sensory processes, emotion and attention regulation, and cognition (Cauda et al., 2012; Uddin, 2015). Importantly, it should be pointed out that the insula is parcellated into two regions: (1) the anterior region, which is connected to the frontal cortex, ACC, parietal, and limbic regions, is mainly involved in salience detection and other emotional aspects; and (2) the posterior region, which is linked to the premotor, sensorimotor, temporal and posterior cingulate regions, is responsible for processing perception, emotion, sensorimotor integration, and interoception (Cauda et al., 2011, 2012). In particular, previous work has revealed that insomnia subjects exhibit insula abnormalities. For example, our previous findings showed decreased fractional ALFF in the left anterior insula and bilateral posterior insula, which can offer an explanation for the sleep state misperception and hyperarousal in insomnia (Liu C. H et al., 2016). Stoffers et al. demonstrated that diminished gray matter density in the left inferior orbitofrontal cortex borders the insula in patients with earlier morning awakening (Stoffers et al., 2012). Huang et al. found that decreased functional connectivity was identified mainly between the amygdala and the insula, striatum and thalamus in patients with insomnia (Huang et al., 2012). In a recent study, Chen et al. discovered that female insomniacs had increased activation of the anterior insula with salience networks, and they further revealed the relationship between blood-oxygen-level dependent (BOLD) in insula and the EEG gamma frequency power (Chen et al., 2014). Li et al. also reported a stronger connectivity between the bilateral superior parietal lobe and the right insular lobe in insomnia (Li et al., 2014). More recently, Wang et al. found an increased regional homogeneity (ReHo) value in the left insula in insomnia patients (Wang et al., 2016). These inconsistent results may be due to the diversity of the sample size of the insomnia subjects, gender differentiation, and methodological difference used. Interestingly, although the present study did not identify an altered topology in the insula in healthy subjects with insomnia, our results on decreased nodal network topology of the IFG were consistent with previous findings that revealed the relationship between altered insula activity and insomnia. More importantly, because the IFG is connected to the anterior insula and the aberrant IFG topology is also identified in insomnia for the present work, we speculated that the IFG may comprise an important biomarker for the hyperarousal pathophysiology of insomnia. Taken together, it is suggested that insomnia may disturb the role of the IFG in the maintenance of alertness and cognitive process functions.

More interestingly, a positive relationship between the nodal betweenness centrality of the right putamen and the insomnia score was also identified, indicating that a stronger putamen centrality control of information flow within the network was associated with more severe insomnia. The putamen is a key component of the striatum and is a critical component of the fronto-striatum circuits as well. Importantly, the insula and thalamus are strongly preferentially associated with the intrinsic coactivation pattern of the right putamen (Postuma and Dagher, 2006; Di Martino et al., 2008). The insula of the salience network plays an essential role in saliency detection, decision-making, motor/sensory processes, emotion and attention regulation, and cognition insomnia. Meanwhile, the thalamus has been confirmed to play crucial roles in various functions, including arousal functions, cognitive functions, affective and reward processing and motor organizations (Saalmann, 2014). In particular, the thalamus is connected to the ventral striatum, amygdala and medial prefrontal cortex, which is implicated in diverse functions, including reward-based decision-making, motivation drive, planning, and monitoring decisions for the development and expression of reward-based behaviors (Akert and Hartmann-von Monakow, 1980; Haber and Calzavara, 2009; Krebs et al., 2012). Moreover, the thalamus is a complex association of central nuclei that are closely correlated and interposed with the arousal and frontal systems, thus linking to the levels of vigilance and wakefulness of sleep (Bridoux et al., 2015). Overall, the insula of the salience network and the thalamus of the hyperarousal system are essential for insomnia, which indicates the hub of the insula and thalamus in sleep-related activity (Spiegelhalder et al., 2015). Using task-based fMRI, Bell-McGinty et al. also discovered increased activation in the right putamen in subjects after total sleep deprivation (TSD), which revealed that changes in the arousal levels may affect performance following TSD (Bell-McGinty et al., 2004). As a result, although the insula did not appear in our mapping results, the altered putamen revealed in this study is of considerable importance in insomnia. According to the identified IFG and putamen, we concluded that the topology of the fronto-striatum connectivity develops distinctively in insomnia. Specifically, our findings demonstrated that the insomnia may either result from, or contribute to the disturbed fronto-striatum network connectivity.

## Limitations

To appropriately access the present findings, several limitations should be discussed. First, the Pittsburgh Sleep Quality Index or Duke structured interview for insomnia or sleep disorders were not adopted in this study, instead, we used the HAMD-17 with its three-item sleep subscale, which was better correlated with sleep diaries (Manber et al., 2005). Second, the functional brain networks in this study were only constructed at a regional level by parcellating the whole brain into 90 regions based on the AAL atlas. Previous studies have revealed that different parcellation strategies can exhibit distinct topological properties. Future studies should plan to systematically examine the most appropriate parcellation strategy to map the brain network topology in insomnia.

## Conclusions

To the best of our knowledge, this is the first study to examine the aberrant topological organization of intrinsic brain networks in insomnia. We discovered that both healthy subjects with and without insomnia symptoms exhibited the small-worldness topology. No significant differences were identified between the IS and NIS groups according to the measures of global topological properties. However, altered regional network properties of the salience and frontostriatal networks were strongly linked with insomnia. Consequently, our findings presented novel evidence that disrupted topological organizations of the frontostriatal connectivity and salience networks may be a cause or consequence of insomnia.

## Author contributions

FL, CL, SL, LT, CT, and ZY conceived and designed the experiments. SL, LT, and CT acquired the data, which FL and ZY analyzed the data. FL, CL, JZ, and ZY wrote the article, which all authors reviewed and approved for submission.

## Funding

This work was supported by MYRG2014-00093-FHS, MYRG2015-00036-FHS and MYRG2016-00110-FHS grants from the University of Macau and FDCT 026/2014/A1 and FDCT 025/2015/A1 grants from Macao government in Macau. This work was also supported by the National Natural Science Foundation of China (grant 81471389) and the High-Level Health Technical Personnel in Beijing (grant 2014-3-095).

### Conflict of interest statement

The authors declare that the research was conducted in the absence of any commercial or financial relationships that could be construed as a potential conflict of interest.
