# Supplementary material for: Disrupted Topology of Frontostriatal Circuits Is Linked to the Severity of Insomnia
Source: Front Neurosci. 2017 Apr 19;11:214. doi: 10.3389/fnins.2017.00214 (PMC5395640; doi:10.3389/fnins.2017.00214)
Supplement: Supplementary file 1 [file Presentation1.pdf]

Supplementary Material

Disrupted topology of frontostriatal circuits is linked to the severity of insomnia

Feng-Mei Lu [1], Chun-Hong Liu [2, 3], Shun-Li Lu [3], Li-Rong Tang [3], Chang-Le Tie [3], Juan Zhang [4] and Zhen Yuan [1]*

*** Correspondence:** Corresponding Author: Prof. Zhen Yuan, zhenyuan@umac.mo

1 Supplementary Methods

Network Analysis

Small-world network parameters

The functional connectivity network can be evaluated using graph theory analysis in which a network comprised the nodes and edges. For an N × N (N = 90 indicates 90 nodes in the present study) binary undirected graph *G*, the topological properties were defined on the basis of the following graph construction:

$$e_{ij} = \begin{cases} 1, & if\ |z_{ij}| \geq T \\ 0, & otherwise \end{cases},$$

If the absolute z_{ij} (the Fisher *r*-to-*z* of the partial correlation coefficient between node i and node j) exceeds a given threshold *T*, an undirected edge is said to exist; otherwise it does not exist.

The clustering coefficient of a node i is defined as the ratio of the number of existing connections among the node's neighbors to the number of all possible connections in the subgraph G_i (Onnela et al., 2005) and is expressed as:

$$C_i = \frac{E_i}{K_i\,(K_i - 1)/2},$$

in which E_i and K_i denote the number of edges and nodes respectively in the subgraph G_i. Then the clustering coefficient of a functional connectivity network is the average of the clustering coefficients of all nodes:

$$C_p = \frac{1}{N}\sum_{i \in G} C_i,$$

it measures the local interconnectivity of a network.

The mean shortest path length of a node i is defined as:

$$L_i = \frac{1}{N-1}\sum_{i \neq j \in G} min|L_{ij}|,$$

where $min|L_{ij}|$ is the absolute shortest path length (i.e. the smallest number of edges traversed between two nodes) between node i and node j. The mean shortest path length of a network is then the average of the shortest path lengths between the nodes:

$$L_p = \frac{1}{N}\sum_{i \in G} L_i,$$

The normalized clustering coefficient $\gamma = \frac{C_p}{C_{random}}$ and normalized characteristic path length $\lambda = \frac{L_p}{L_{random}}$ were computed, where C_p and L_p indicate the mean clustering coefficient and shortest path length of the functional connectivity network, respectively. C_{random} and L_{random} represent the mean clustering coefficient and shortest path length of 100 matched random networks that preserved the same number of nodes, edges, and degree distribution as the real network (Sporns and Zwi, 2004; Ding et al., 2011). Typically, a small-word network meet the conditions of $\gamma > 1$ and $\lambda \approx 1$, and therefore, the small-wordness scalar $\sigma = {}^{\lambda}/_{\gamma}$ will be more than 1.

Efficiency of small-world networks

Network efficiency can be measured by global efficiency, E_{glo}, local efficiency, E_{loc}

and nodal efficiency, E_{nodal}, E_{glo} and E_{loc} described the ability of information transmission of a network at the global and local level, respectively. The global efficiency E_{glo} of a network is the inverse of the harmonic mean of the shortest path length between each pair of nodes (Latora and Marchiori, 2001; Achard and Bullmore, 2007):

$$E_{glo} = \frac{1}{N(N-1)} \sum_{i \neq j \in G} \frac{1}{min|L_{ij}|},$$

where $min|L_{ij}|$ is the absolute shortest path length between node i and node j in network *G.* It indicates the capability of parallel information transfer through the whole network.

The nodal efficiency E_{nodal} of a node i is calculated as:

$$E_{nodal}(\mathrm{i}) = \frac{1}{N-1} \sum_{j,k \in G} \frac{1}{min|L_{jk}|},$$

The local efficiency E_{loc} denoted the mean of all the local efficiencies of the nodes in subgraph G_i which is defined as:

$$E_{loc} = \frac{1}{N} \sum_{i \in G} E_{nodal}(i),$$

where $E_{nodal}(i) = E_{glo}(G_i)$. Since the node i is not an element of the subgraph G_i, the local efficiency can also be considered as a measure of the fault tolerance of the network, suggesting how well each subgraph exchanges information when the node i was eliminated (Achard and Bullmore, 2007).

The integrated area under curve (AUC) of a network metric Y was computed over the sparsity threshold range from S_1 to S_n with interval of ΔS, which was expressed as:

$$Y^{AUC} = \sum_{k=1}^{n-1} [Y(S_k) + Y(S_{k+1})] \times \Delta S/2.$$

2 Supplementary Tables

Table S1.

Anatomical regions of interest (ROIs) and abbreviated regional labels.

**Region name**	**Abbr.**	**Region name**	**Abbr.**
Precentral gyrus	PreCG	Lingual gyrus	LING
Superior frontal gyrus	SFG	Superior occipital gyrus	SOG
Superior frontal gyrus, orbital	SFGorb	Middle occipital gyrus	MOG
Middle frontal gyrus	MFG	Inferior occipital gyrus	IOG
Middle frontal gyrus, orbital	MFGorb	Fusiform gyrus	FG
Inferior frontal gyrus, opercular part	IFGoper	Postcentral gyrus	PoCG
Inferior frontal gyrus, triangular	IFGtri	Superior parietal gyrus	SPG
Inferior frontal gyrus, orbital	IFGorb	Inferior parietal gyrus	IPG
Rolandic operculum	ROL	Supramarginal gyrus	SMG
Supplementary motor area	SMA	Angular gyrus	ANG
Olfactory cortex	OLF	Precuneus	PCUN
Superior frontal gyrus, medial	SFGmed	Paracentral lobule	PCL
Superior frontal gyrus, medial orbital	SFGmorb	Caudate nucleus	CAU
Gyrus rectus	REG	Putamen	PUT
Insula	INS	Pallidum	PAL
Anterior cingulate gyri	ACC	Thalamus	THA
Median cingulate gyri	MCC	Heschl gyrus	HES
Posterior cingulate gyrus	PCC	Superior temporal gyrus	STG
Hippocampus	HIP	Superior temporal gyrus: temporal pole	STGp
Parahippocampal gyrus	PHIP	Middle temporal gyrus	MTG
Amygdala	AMYG	Middle temporal gyrus: temporal pole	MTGp
Calcarine fissure	CAL	Inferior temporal gyrus	ITG
Cuneus	CUN		

The regions are listed according to a prior AAL atlas (Tzourio-Mazoyer et al., 2002).

Abbr., abbreviations.

Table S2.

Introduction of topological properties in the brain functional network.

Properties	Descriptions
Global network properties	
C_p	Clustering coefficient of a network which measures the local interconnectivity of a network. It is the average of the clustering coefficients over all nodes.
L_p	Path length of a network which quantified the level of overall routing efficiency of a network. It is the mean minimum number of connections between any two nodes in the network.
E_{glo}	Global efficiency of a network which indicates the capability of parallel information transfer through the whole network. It is the inverse of the harmonic mean of the minimum path length between any two nodes in the network.
E_{loc}	Local efficiency of a network which captures the fault tolerance of a network. It is the average of the local efficiency over all nodes.
Local network properties	
Deg_i	Nodal degree which evaluates the extent to which the node is connected to the rest of other nodes in a network.
E_{nodal}	Nodal local efficiency which measures the level of information propagation of a node with all other nodes in the network.
BC_i	Betweenness which estimates the influence of a node over information flow with the rest of the nodes in a network.

Reference

Achard, S., and Bullmore, E. (2007). Efficiency and cost of economical brain functional networks. *PLoS Comput Biol* 3(2), e17. doi:10.1371/journal.pcbi.0030017.

Ding, J.R., Liao, W., Zhang, Z., Mantini, D., Xu, Q., Wu, G.R., et al. (2011). Topological fractionation of resting-state networks. *PLoS One* 6(10), e26596. doi: 10.1371/journal.pone.002659.

Latora, V., and Marchiori, M. (2001). Efficient behavior of small-world networks. *Phys Rev Lett* 87(19), 198701. doi:10.1103/physrevlett.87.198701.

Onnela, J.P., Saramaki, J., Kertesz, J., and Kaski, K. (2005). Intensity and coherence of motifs in weighted complex networks. *Phys Rev E Stat Nonlin Soft Matter Phys* 71(6 Pt 2), 065103. doi: 10.1103/PhysRevE.71.065103.

Sporns, O., and Zwi, J.D. (2004). The small world of the cerebral cortex. *Neuroinformatics* 2(2), 145-162. doi:10.1385/NI:2:2:145.

Tzourio-Mazoyer, N., Landeau, B., Papathanassiou, D., Crivello, F., Etard, O., Delcroix, N., et al. (2002). Automated anatomical labeling of activations in SPM using a macroscopic anatomical parcellation of the MNI MRI single-subject brain. *Neuroimage* 15(1), 273-289. doi: 10.1006/nimg.2001.0978.
